# Solid Fat Replacement with Canola Oil-Carnauba Wax Oleogels for Dairy-Free Imitation Cheese Low in Saturated Fat

**DOI:** 10.3390/foods10061351

**Published:** 2021-06-11

**Authors:** Kyungwon Moon, Kyeong-Ok Choi, Sungmin Jeong, Young-Wan Kim, Suyong Lee

**Affiliations:** 1 Department of Food Science and Biotechnology and Carbohydrate Bioproduct Research Center, Sejong University, Seoul 05006, Korea; kwon222@nate.com (K.M.); rlalf3@naver.com (S.J.); 2 Fruit Research Division, National Institute of Horticultural and Herbal Science, Rural Development Administration, Wanju 55365, Korea; ko1786@korea.kr; 3 Department of Food Science and Biotechnology, Korea University (Sejong), Sejong 30019, Korea; ywankim@korea.ac.kr

**Keywords:** fat replacement, oleogelation, rheology, time-domain NMR

## Abstract

Canola oil was structured into oleogels with different amounts of carnauba wax, and their processing performances were assessed as an alternative to solid fat for imitation cheese low in saturated fat. The contents of solid fat in the oleogels were less vulnerable to the change in temperature than the palm oil. The replacement of palm oil with oleogels produced cheese samples with harder and more cohesive/chewy textures. Dynamic and transient viscoelastic measurements demonstrated that the use of oleogels was effective in increasing the elastic nature of the cheeses. Two distinct components with different proton mobilities were observed in the imitation cheeses, and longer T_2_ relaxation times were detected in the oleogel samples. The meltability of the cheese with palm oil was not significantly different from those with 3% and 6% oleogels. The saturated fat level of the oleogel cheese was significantly reduced from 45.70 to 5.20%. The application of canola oil-carnauba wax oleogels could successfully produce imitation cheese high in unsaturated fat and low in saturated fat. This study thus demonstrated that the health-functional properties of imitation cheese could be enhanced by using oleogels.

## 1. Introduction

Vegan foods have recently become a major trend [1] in the global food market for several reasons such as health and ethical issues [2], and the demands for dairy-free products are increasing rapidly around the world. Accordingly, a concerted effort has been made by many food manufacturers to develop dairy-free vegan food products. Out of the various dairy products, a great deal of effort has been made to develop dairy-free imitation cheeses with vegetable ingredients. Specifically, plant-based proteins from soybeans and nuts are widely used in the partial or total replacement of caseinate. Guirguis and El-Neshawy [3] and Oyeyinka et al. [4] utilized peanut protein isolate and cashew nut milk to manufacture cheese analogs, respectively. In preceding studies, soy protein has been extensively used to produce imitation cheese as a main nondairy substitute for milk protein [5,6]. Ahmad et al. [7] reported that soy cheese, known as fermented soybean curd, was similar to milk cheese in many aspects, including physical appearance and texture. In addition, dairy-free imitation cheese was produced through the combination of soybean and glucono-δ-lactone (GDL) [8] and soy protein concentrate [9]. In addition, Omrani Khiabanian et al. [10] used pea protein isolate instead of milk protein in feta cheese. However, the use of plant-based proteins in cheese brought about processing difficulties in mimicking the desirable cheese texture and meltability generated by caseinates [6]. Therefore, food ingredients such as hydrocolloids [11] and starches [12] have been incorporated as texture modifiers into the formulation of cheese analogs.

Cheese is recognized as a great source of protein but is high in saturated fat. Likewise, most dairy-free imitation cheeses have a high level of saturated fat, as palm oil has been typically used as a lipid component [13]. Although palm oil is a plant-derived oil, it has a high content of saturated fat, the overconsumption of which has been linked to adverse health effects such as cardiovascular disease [14]. Thus, the World Health Organization (WHO) recommends reducing the intake of saturated fat [15]. A great deal of effort has been made to reduce the content of fat in cheeses. McMahon et al. [16] utilized protein- and carbohydrate-based fat replacers for low-fat mozzarella cheese. Additionally, inulin has previously been used in replacing fat for cheese [17]. Likewise, several researchers have attempted to replace fat in imitation cheeses. Noronha et al. [18] and Liu et al. [19] applied resistant starch and pectin gel as fat alternatives to reduce the fat level of cheese analogs, respectively. However, because most of the fat replacers previously tested have different chemical structures from conventional fat, they cannot replace fat fully on a one-to-one basis, potentially deteriorating the quality attributes of cheese [20]. There is, therefore, a need to apply suitable vegetable lipids low in saturated fat that can provide the same quality attributes the original solid fats would provide.

Oleogelation is an innovative technology that structures liquid vegetable oil into a gel-like material [21]. Through oleogelation, liquid vegetable oil can maintain a self-standing solid structure, although it contains a lot of unsaturated fat. Oleogelation is essential for solidifying liquid oil, and various materials such as natural waxes, fatty acids, polymer agents, and emulsifiers have been utilized to generate oleogels [22,23,24,25]. Among the diverse oleogelators, waxes such as rice bran wax and carnauba wax have been extensively used in a number of preceding studies because of the advantages of affordable cost, availability, and being Generally Recognized as Safe (GRAS) [26,27]. More recently, many studies have reported the use of oleogel as a substitute for solid fat high in saturated fatty acids in various food products such as biscuits [28], meat [29], chocolate, spread [30], and ice cream [31]. However, preceding studies to utilize oleogels as a solid fat substitute in imitation cheeses have not been carried out to the best of our knowledge.

Therefore, in this study, oleogels were prepared by structuring canola oil with carnauba wax as an oleogelator and applied as substitutes for palm oil in producing dairy-free imitation cheese low in saturated fatty acids. The effects of palm oil replacement with oleogels on the physicochemical characteristics of the imitation cheese were evaluated mainly in terms of rheological and proton mobility properties.

## 2. Materials and Methods

### 2.1. Preparation of Canola Oil Oleogels

According to the methods of Jung et al. [32], oleogels were prepared with canola oil (Sajo Haepyo Co. Ltd., Seoul, Korea) and carnauba wax (Starlight Co., Fortaleza, Brazil). The canola oil was heated to 90 °C in a beaker and blended with carnauba wax until completely dissolved during agitation on a laboratory stirrer (200 rpm). The blending ratios were 97:3 (3% oleogel), 94:6 (6% oleogel), and 91:9 (9% oleogel) by weight. The resultant mixture was then cooled to room temperature for 1 h and placed in a refrigerator for further use.

### 2.2. Determination of Solid Fat Content

Based on the approved methods of AOCS [33], the solid fat contents of palm oil (Lotte Food Co. Ltd., Seoul, Korea) and oleogels were analyzed using time-domain nuclear magnetic resonance (TD-NMR) (MQC+, Oxford Instruments, Oxon, UK) at temperatures from 10 to 90 °C at an interval of 10 °C. Each sample (≈2 g) was loaded in an NMR glass tube (10 mm diameter) and placed at 100 °C for 15 min, 60 °C for 10 min, and then 4 °C for 1 h. Afterward, the tube was maintained at given measuring temperatures for 30 min and then loaded into the NMR.

### 2.3. Preparation of Imitation Cheese

The formulation of the control imitation cheese consisted of 38.0 g of soy protein isolate (SPI) (Sungpoong Co., Anseong, Korea), 341.8 g of water, 3.8 g of salt (CJ Co., Seoul, Korea), 22.8 g of tapioca starch (Heungyil, Seoul, Korea), 15.2 mL of lemon juice (Kaya Korea, Seoul, Korea), 13.5 g of agar powder (Ehomebakery, Incheon, Korea), 24.0 g of sugar (Samyang Co., Seoul, Korea), and 51.0 g of palm oil. The agar powder with water was agitated at 200 rpm for 10 min by using a stirrer (WiseStir, Daihan Scientific, Wonju, Korea). The sugar was then added and mixed for 1 min. This mixture was heated on a hot plate (Kitchenflower Co., Gimpo, Korea) for 2 min, and the palm oil was then added, followed by agitation for 1 min. Afterward, SPI, water, salt, tapioca starch, and lemon juice were added to the mixture and agitated with heating for 8 min. The resulting sample was poured into a plastic container (length = 130 mm, width = 90 mm, height = 60 mm), cooled to room temperature for 30 min, and then stored in a refrigerator overnight. In the case of the oleogel samples, the palm oil was replaced with three different oleogels (3, 6, and 9%) on an equal weight basis.

### 2.4. Color Measurement

The color properties of the imitation cheese samples with palm oil and oleogels were investigated by using a colorimeter (ColorFlex EZ 45/0, HunterLab, Reston, VA, USA). Their surface color values L (lightness/darkness), a (redness/greenness), and b (yellowness/blueness) were recorded.

### 2.5. Texture Measurement

A texture analyzer (TA-XT plus, Stable Micro System Ltd., Surrey, UK) was applied to measure the textural properties of the imitation cheese samples prepared with palm oil and oleogels. The cheese samples were cut into cubic shapes (17 × 17 × 17 mm) and subjected to texture profile analysis (a 50 mm diameter cylindrical probe, 60% strain, and 100 mm/min crosshead speed).

### 2.6. Viscoelastic Measurement

The viscoelastic properties of the cheese samples with palm oil replacement with oleogels were investigated in two different ways: dynamic oscillatory and stress relaxation tests. The dynamic viscoelastic measurements were made at 25 °C using a Discovery HR-2 rheometer (TA Instrument, New Castle, DE, USA) with a 40 mm diameter hatch parallel plate. The frequency sweep test was carried out in the frequency range of 0.1 to 10 Hz within the linear viscoelastic limit (0.1% strain).

Based on the methods of Lim et al. [34], each cheese specimen was cut into a cubic piece (17 × 17 × 17 mm) and then subjected to a stress relaxation test using a texture analyzer (TA-XT plus, Stable Micro System Ltd., Surrey, UK) equipped with a cylindrical probe (50 mm diameter). They were instantaneously compressed to a 30% strain, and the deformation was held constant for 3 min. The stress relaxation curves were monitored and fitted in order to obtain the stress relaxation parameters.

### 2.7. T_2_ Relaxation Time Analysis by Proton NMR

The proton mobilities of the cheese samples with palm oil and oleogels were measured using TD-NMR (23.4 MHz, MQC+, Oxford Instruments, Oxon, UK). Each cheese sample was loaded in an NMR tube (18 mm diameter) and subjected to the Carr–Purcell–Meiboom–Gill (CPMG) pulse sequence to measure its spin–spin (T_2_) relaxation time at 40 °C. The average number of signal acquisitions was 32 scans, tau was 121.5 μs, and the number of echoes was 4096. The CPMG relaxation curves obtained were fitted as a continuous distribution of exponentials with WinFit software from Oxford Instruments.

### 2.8. Determination of Meltability

Based on the method of Mounsey and O’Riordan [35], a cube-shaped piece of cheese (17 × 17 × 17 mm) was loaded into a glass tube (30 mm diameter × 200 mm height), which was horizontally placed in a dry oven (OF-12GW, Jeio tech. Co. Ltd., Daejeon, Korea) at 180 °C for 10 min. The tube was then removed from the oven, and the distance flowing (mm) was used as an indicator of meltability.

### 2.9. Determination of Fatty Acid Composition

A gas chromatograph (GC) (6890N, Agilent Technologies, Santa Clara, CA, USA) equipped with an Agilent 5975 series mass selective detector was applied to analyze the fatty acid compositions of the cheese samples prepared with palm oil and oleogels. After the cheese was subjected to Soxhlet extraction with diethyl ether [36], fatty acid methyl ester (FAME) derivatives prepared with KOH methanol and 10% BF3-methanol (Sigma-Aldrich, St. Louis, MO, USA) were separated on an HP-INOWAX capillary column (30 m × 0.32 mm × 0.25 μm, Agilent Technologies), and the flow rate of purified helium carrier gas was 2 mL/min. The injector temperature was 300 °C, and the column temperature was maintained at 100 °C for 5 min that was then heated to 250 °C (3 °C/min) and held for 5 min. The National Institute of Standards and Technology (NIST) 11 mass spectral library (NIST 11) was used for identifying fatty acids in the mass spectral results obtained.

### 2.10. Statistical Analysis

Three batches of each imitation cheese formulation were prepared, and the reported values were expressed as the mean ± standard deviation. The R statistical package (The R Foundation for Statistical Computing, Vienna, Austria) was used to statistically analyze experimental results. Analysis of variance was carried out, followed by Duncan’s multiple range test for comparisons (95%).

## 3. Results and Discussion

### 3.1. Determination of Solid Fat Content

**Figure 1:**
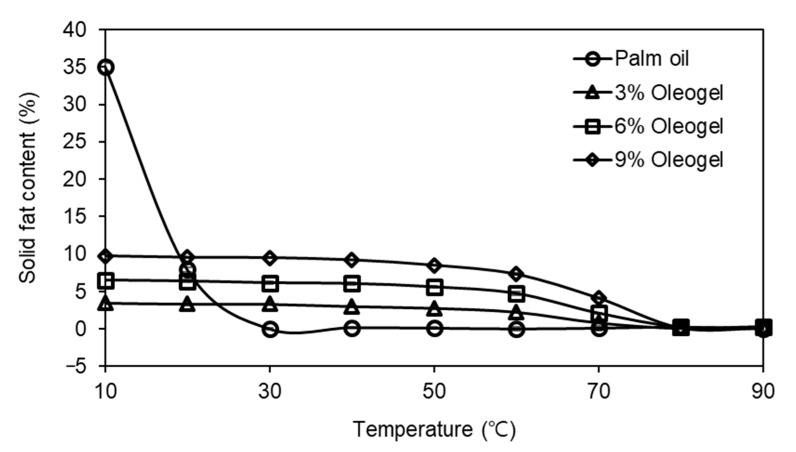
Solid fat content of palm oil and oleogels.

The palm oil and oleogel samples exhibited different profiles of solid fat contents over temperature (Figure 1). The highest solid fat content (35%) was observed in the palm oil at 10 °C. In the case of the oleogels, their solid fat contents were determined to be 3, 6, and 9% at 10 °C, showing higher solid fat contents with increasing contents of oleogelator. As clearly shown in Figure 1, the solid fat content of the palm oil sharply decreased in the temperature range from 10 to 30 °C, and this pattern was in good agreement with the results reported by Aini and Miskandar [37], while little changes in the solid fat content values were observed in the oleogel samples at a temperature lower than 50 °C and then started to decrease by melting. Thus, the solid fat content of the palm oil was more sensitive to temperature, compared to the oleogel samples. This temperature dependence of the oleogels prepared with carnauba wax was in great agreement with the results by Yi et al. [38].

### 3.2. Color Measurement

The color parameters (L, a, and b) of the imitation cheese samples are presented in Table 1, where the values of L, a, and b indicate light/dark, red/green, and yellow/blue colors, respectively [39]. The lightness (L value) of the oleogel cheese samples was significantly lower than that of the palm oil cheese, resulting in a slightly darker color. The L values tended to slightly increase with increasing levels of oleogelator. However, there were no significant differences in the values among the samples. In the case of b values, they seemed to increase in the imitation cheese samples prepared with oleogels. These color changes of the imitation cheeses could be derived from the intrinsic dark and yellow color of the oleogels prepared with canola oil and carnauba wax, compared to palm oil (L: 61.37–67.07 and b: 16.47–24.11 for oleogels, L:73.31 and b:15.75 for palm oil, data not shown).

### 3.3. Texture Analysis

Texture is a critical factor affecting the sensory quality and consumer acceptance of cheese [40]. Thus, the texture properties of the imitation cheese samples made from palm oil and oleogels were compared, as shown in Table 2. For all the samples, the first significant break in the curve that is a measure of fracturability [41] was observed, and its force values tended to increase with increasing levels of oleogelator. As presented in Table 2, the use of oleogels for palm oil replacement produced the imitation cheese samples with a harder texture. More cohesive and chewy textures were also observed in the oleogel samples. However, there were no apparent differences in the adhesiveness and springiness parameters among the samples. Guinee, et al. [42] reported several undesirable texture properties of the cheese analogs prepared with plant-based protein such as low hardness and high adhesiveness. However, it seemed that the use of oleogels positively contributed to controlling the cheese texture. It was reported that the texture of products with oleogels was affected by the solid fat content of the oleogels incorporated [43]. Hence, these textural changes of the imitation cheeses by the use of oleogels could be expected from higher solid fat contents of the oleogels prepared with higher amounts of carnauba wax at room temperature.

### 3.4. Dynamic Viscoelastic Measurement

**Figure 2:**
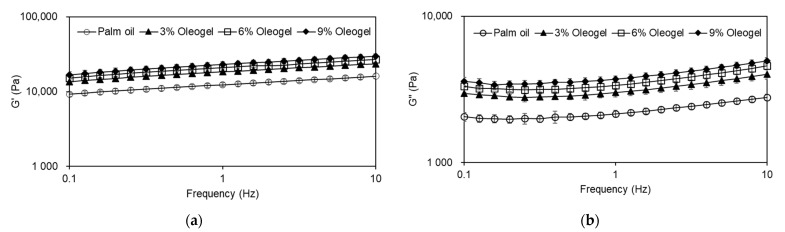
Effect of palm oil replacement with oleogels on the dynamic viscoelastic characteristics of imitation cheeses: (**a**) G’ (Storage module); (**b**) G’’ (loss module).

It is well recognized that cheese is a viscoelastic food with liquid- and solid-like features [40]. Therefore, the dynamic viscoelasticity of the cheeses made from palm oil or oleogels was characterized, as shown in Figure 2. The G’ values were higher than the G’’ values in all the cheese samples within the frequency range tested, meaning that more energy was stored rather than dissipated. In addition, G’ and G’’ values tended to be frequency dependent with consistent, positive slopes, while a plateau modulus was not achieved. Therefore, all of the imitation cheese samples made from palm oil and oleogels possessed weak gel-like characteristics. When the palm oil was replaced with oleogels, the G’ and G’’ values of the imitation cheese distinctly increased. Moreover, the use of the oleogels with a higher proportion of oleogelator led to an increase in the viscoelastic properties of the imitation cheese samples.

### 3.5. Transient Viscoelastic Measurement

In addition to the dynamic viscoelastic responses, the stress relaxation behaviors of the cheese samples were evaluated. The stress relaxation curves were satisfactorily (R^2^ ≥ 0.9996) fitted to the following equation [44].

(1) $$\frac{\left(F_{0}\times t\right)}{\left(F_{0}-F\left(t\right)\right)}=k_{1}+k_{2}\times t$$

where *F_o_* is the initial force, *F*(*t*) is the force at a time of *t*, %SR is F_max_/*F*(*t*) × 100, RT is the time required for the stress to drop to 36.8% of the initial value, k_1_ is the intercept, and k_2_ is the slope.

As presented in Table 3, higher values of Fmax were clearly observed in the oleogel-incorporated cheeses, compared to that with palm oil. In addition, the %SR was close to 100% in the elastic material and became higher in the cheese samples with higher levels of oleogelator. In the case of relaxation time (RT), it showed a distinct tendency to increase in the oleogel samples. In the stress relaxation test, the stress in liquids relaxes quicker than that in solids due to the higher mobility of liquid molecules, suggesting that the relaxation time is short for liquids, while it is long for elastic solids [41]. These results were consistent with higher k_1_ and k_2_ values of the oleogel samples, which indicates the initial rate and extent of relaxation [44], respectively. Thus, the use of oleogels instead of palm oil contributed to the increased elasticity of the cheese samples, which was in great agreement with the dynamic viscoelastic results (Figure 2).

### 3.6. Determination of T_2_ Relaxation Times

**Figure 3:**
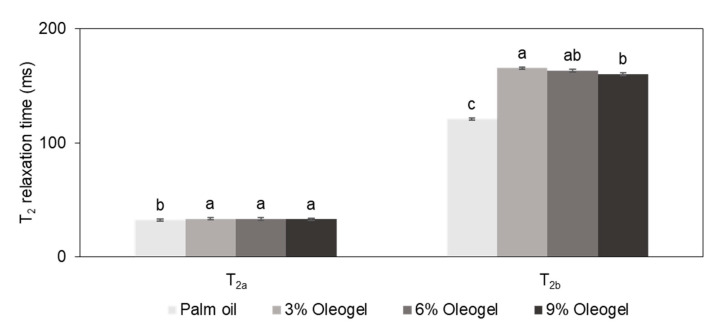
Effect of palm oil replacement with oleogels on the spin–spin relaxation (T2) times of imitation cheeses (means with different letters in the same classification significantly differ at *p* < 0.05).

Figure 3 exhibits the T_2_ relaxation times of the imitation cheese samples prepared with palm oil and oleogels. In the time-domain NMR analysis, the T_2_ relaxation times indicate the mobility of a component [45]. Two distinct components with different relaxation times (T_2a_ and T_2b_) were clearly observed, showing good agreement with the results of Budiman et al. [46] and El-Bakry et al. [47], who measured the T_2_ relaxation times of cheese analogs depending on the type of fat and moisture content, respectively. The relaxation times of T_2a_ and T_2b_ were in the range of 30–33 and 120–165 ms, respectively. It seemed that the T_2a_ component with a short relaxation time corresponded to tightly bound water, while the oil in the cheese contributed to the T_2b_ component. The changes in the T_2a_ relaxation times were marginal among the samples, although a significant difference was observed between the palm oil and oleogel cheese samples. In the case of T_2b_, the cheese with palm oil had a T_2b_ relaxation time of 120.91 ms, which was consistent with that (126.67 ms) in the imitation cheese reported by Noronha et al. [48]. For the oleogel cheese samples, their T_2b_ relaxation times had a tendency to decrease with increasing levels of oleogelator, suggesting that the mobility of oil might be restricted as expected from high contents of solid fat in Figure 1.

### 3.7. Determination of Cheese Meltability

The effect of palm oil replacement with oleogels on the imitation cheese meltability was evaluated. As presented in Table 4, the meltability value of the cheese made from palm oil was determined to be 82.75 mm, which was not significantly different from the cheese samples with 3% and 6% oleogels. However, the use of 9% oleogel gave rise to the decreased melting properties of the imitation cheese. Carnauba wax is constituted of a complex mixture that primarily consists of aliphatic esters [49]. Among the wax-based oleogelators that can be used in food, carnauba wax is considered as a natural wax with a hard texture and the highest melting point [50]. Therefore, the higher proportion of carnauba wax at more than 9% (*w/w*) in the oleogels might have a slightly negative effect on the cheese meltability.

### 3.8. Analysis of Fatty Acid Composition

Table 5 shows the fatty acid compositions of the imitation cheese samples. The cheese made from palm oil was high in palmitic acid (C16:0), oleic acid (C18:1), and linoleic acid (C18:2), and its saturated fat content was determined to be 45.7%. In the case of the oleogel cheeses, the primary fatty acids were oleic acid (C18:1), linoleic acid (C18:2), and linolenic acid (C18:3) that, together, accounted for approximately 94% of total fatty acids. These compositions could be favorably compared with the results reported by Jang et al. [51] and Kim et al. [52] that analyzed the fatty acid compositions of canola oil and baked goods prepared with canola oil–carnauba wax oleogels, respectively. Therefore, the use of oleogel for palm oil was very effective in lowering the ratio of saturated and unsaturated fat from 0.84 to 0.06, successfully producing the imitation cheese samples high in unsaturated fat and low in saturated fat.

## 4. Conclusions

Canola oil and carnauba wax were used to prepare oleogels that were utilized as an alternative to palm oil in order to reduce the level of saturated fat in dairy-free imitation cheese products. The replacement of palm oil with oleogels positively contributed to the textural and rheological properties of the imitation cheese samples by enhancing their elastic nature. Additionally, the use of 3 and 6% oleogels for palm oil did not affect the cheese meltability. Furthermore, the cheese samples containing oleogels showed nutritional superiority by significantly lowering the ratio of saturated and unsaturated fat from 0.84 to 0.06. As the first research on the application of oleogels to imitation cheese, this study could provide fundamental results related to the processing performance of oleogels in dairy-free imitation cheese. In a further study, sensory evaluation will be necessary to evaluate consumer preference and acceptance for practical food applications.

## Figures and Tables

**Table 1:** Visual appearance and color of the imitation cheeses prepared with palm oil and oleogels.

	Palm Oil	3% Oleogel	6% Oleogel	9% Oleogel
Visual appearance	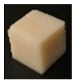	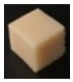	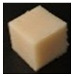	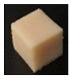
L	71.95 ± 0.11 a	68.04 ± 0.19 d	69.18 ± 0.15 c	69.81 ± 0.26 b
a	−0.26 ± 0.04 a	−0.30 ± 0.03 a	−0.26 ± 0.01 a	−0.25 ± 0.04 a
b	14.55 ± 0.05 c	14.25 ± 0.13 d	14.88 ± 0.09 b	15.11 ± 0.04 a

Means with different letters in the same row differ significantly at *p* < 0.05.

**Table 2:** Effect of palm oil replacement with oleogels on the texture properties of imitation cheeses.

	Palm Oil	3% Oleogel	6% Oleogel	9% Oleogel
Fracturability (N)	6.88 ± 0.56 c	8.88 ± 0.36 b	9.79 ± 0.90 a	9.94 ± 0.57 a
Hardness (N)	7.06 ± 0.31 d	8.09 ± 0.21 c	9.36 ± 0.31 b	9.99 ± 0.29 a
Adhesiveness (N·mm)	1.60 ± 0.55 a	1.06 ± 0.42 a	1.55 ± 0.55 a	1.48 ± 0.69 a
Springiness	0.32 ± 0.03 a	0.33 ± 0.06 a	0.35 ± 0.05 a	0.36 ± 0.05 a
Cohesiveness	0.14 ± 0.01 b	0.14 ± 0.01 b	0.15 ± 0.01 b	0.17 ± 0.01 a
Chewiness (N·mm)	0.57 ± 0.11 b	0.60 ± 0.10 b	0.82 ± 0.15 a	0.99 ± 0.23 a

Means with different letters in the same row differ significantly at *p* < 0.05.

**Table 3:** Effect of palm oil replacement with oleogels on the stress relaxation parameters of imitation cheeses.

	Palm Oil	3% Oleogel	6% Oleogel	9% Oleogel
F_max_ (N)	8.43 ± 1.05 b	9.95 ± 0.65 a	10.15 ± 0.90 a	10.26 ± 0.46 a
%SR	25.97 ± 3.17 c	28.54 ± 1.42 bc	29.49 ± 2.11 b	34.24 ± 2.23 a
RT (sec)	9.07 ± 2.19 c	10.99 ± 1.20 bc	12.54 ± 1.77 b	18.11 ± 3.76 a
k_1_ (sec)	3.34 ± 0.64 c	3.95 ± 0.31 bc	4.44 ± 0.34 ab	4.93 ± 0.49 a
k_2_	1.21 ± 0.03 b	1.22 ± 0.02 b	1.23 ± 0.03 b	1.30 ± 0.03 a
R^2^	0.9998	0.9998	0.9996	0.9997

Means with different letters in the same row differ significantly at *p* < 0.05.

**Table 4:** Effect of palm oil replacement with oleogels on the meltability of imitation cheeses.

	Palm oil	3% Oleogel	6% Oleogel	9% Oleogel
Meltability (mm)	82.75 ± 1.26 a	82.25 ± 0.50 a	80.50 ± 1.29 ab	78.50 ± 2.38 b

Means with different letters in the same row differ significantly at *p* < 0.05.

**Table 5:** Effect of palm oil replacement with oleogels on the fatty acid composition of imitation cheeses.

Fatty Acid (%)	Palm Oil	3% Oleogel	6% Oleogel	9% Oleogel
C12:0	0.25 ± 0.03	-	-	-
C14:0	0.94 ± 0.12	-	-	-
C16:0	40.65 ± 0.44 a	3.15 ± 0.81 c	4.22 ± 0.67 b	3.90 ± 0.34 bc
C18:0	3.86 ± 0.14 a	1.34 ± 0.06 b	1.30 ± 0.14 b	1.42 ± 0.12 b
C22:0	-	0.35 ± 0.07 a	0.46 ± 0.05 a	0.42 ± 0.09 a
C24:0	-	0.36 ± 0.05 b	0.51 ± 0.07 ab	0.43 ± 0.07 ab
C16:1	-	0.05 ± 0.01 a	0.06 ± 0.02 a	0.06 ± 0.04 a
C18:1	42.72 ± 0.44 c	61.79 ± 1.13 a	60.35 ± 0.22 b	60.43 ± 0.27 b
C18:2	11.38 ± 0.09 c	22.60 ± 0.51 b	23.07 ± 0.10 a	22.97 ± 0.06 ab
C18:3	-	9.97 ± 0.29 a	9.61 ± 0.23 a	9.81 ± 0.13 a
C20:4	0.20 ± 0.02 c	0.39 ± 0.08 b	0.43 ± 0.04 b	0.56 ± 0.08 a
SFA	45.70 ± 0.47 a	5.20 ± 0.92 c	6.48 ± 0.52 b	6.17 ± 0.18 b
USFA	54.30 ± 0.47 c	94.80 ± 0.92 a	93.52 ± 0.52 b	93.83 ± 0.18 b
SFA/USFA	0.84 ± 0.02 a	0.06 ± 0.01 b	0.07 ± 0.01 b	0.07 ± 0.01 b

Means with different letters in the same row differ significantly at *p* < 0.05 (‘-’: not detected).

## Data Availability

Not applicable.
